# Effects of Dietary Strategies on Exercise-Induced Oxidative Stress: A Narrative Review of Human Studies

**DOI:** 10.3390/antiox10040542

**Published:** 2021-03-31

**Authors:** Zhen Zeng, Christoph Centner, Albert Gollhofer, Daniel König

**Affiliations:** 1 Department of Sport and Sport Science, University of Freiburg, 79117 Freiburg, Germany; christoph.centner@sport.uni-freiburg.de (C.C.); ag@sport.uni-freiburg.de (A.G.); 2 School of Sports Medicine and Health, Chengdu Sport University, Chengdu 610041, China; 3 Praxisklinik Rennbahn, 4132 Muttenz, Switzerland; 4 Centre of Sports Science, Department for Nutrition, Exercise and Health, University of Vienna, 1150 Vienna, Austria; daniel.koenig@univie.ac.at; 5 Faculty of Life Sciences, Department for Nutrition, Exercise and Health, University of Vienna, 1090 Vienna, Austria

**Keywords:** diet, antioxidants, exercise, oxidative stress, reactive oxygen species

## Abstract

Exhaustive exercise can induce excessive generation of reactive oxygen species (ROS), which may enhance oxidative stress levels. Although physiological levels are crucial for optimal cell signaling and exercise adaptations, higher concentrations have been demonstrated to damage macromolecules and thus facilitate detrimental effects. Besides single dosages of antioxidants, whole diets rich in antioxidants are gaining more attention due to their practicality and multicomponent ingredients. The purpose of this narrative review is to summarize the current state of research on this topic and present recent advances regarding the antioxidant effects of whole dietary strategies on exercise-induced oxidative stress in humans. The following electronic databases were searched from inception to February 2021: PubMed, Scope and Web of Science. Twenty-eight studies were included in this narrative review and demonstrated the scavenging effects of exercise-induced ROS generation, oxidative stress markers, inflammatory markers and antioxidant capacity, with only one study not confirming such positive effects. Although the literature is still scarce about the effects of whole dietary strategies on exercise-induced oxidative stress, the majority of the studies demonstrated favorable effects. Nevertheless, the protocols are still very heterogeneous and further systematically designed studies are needed to strengthen the evidence.

## 1. Background

The term oxidative stress is defined as a disturbance in the homeostatic balance between pro-oxidants and antioxidants with a subsequent excessive generation of free radicals [1,2,3]. Free radicals are highly reactive compounds that contain one or more unpaired electrons in their outer atomic or molecular orbital [1,4], and thus readily react with various organic substrates in order to make themselves more stable [3]. Species derived from oxygen are generally referred to as reactive oxygen species (ROS) and are naturally occurring byproducts of the human metabolism. Thereby, redox reactions represent fundamental components of organic and biological chemistry [5]. While low to moderate ROS concentrations seem to be involved in cell signaling and muscle remodulation [5,6,7], prolonged exposure to high doses of ROS induces oxidative damage [3]. In case of an insufficient ROS scavenging by antioxidants, high ROS concentrations can lead to modification and damage of cellular molecules including deoxyribonucleic acid (DNA), proteins or lipids [2]. Previous studies have also shown that oxidative stress is involved in the pathophysiology of a wide range of chronic diseases including cancer [5,8], cardiovascular [3,9] and neurological diseases [10,11,12,13]. 

**Figure 1:**
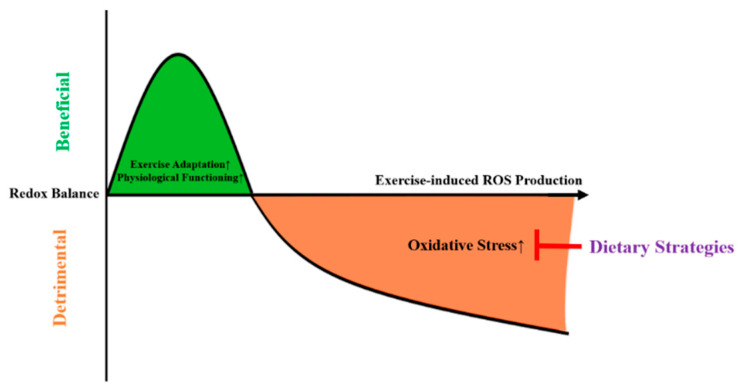
The mitohormesis-based model to explain the effects of dietary strategies on exercise-induced oxidative stress. ROS = reactive oxygen species.

During exercise, the amount of generated ROS seems to be intensity-dependent, with higher exercise intensities leading to supraphysiological ROS formations [14,15]. Mitochondrial hormesis (mitohormesis) was proposed to describe that sublethal mitochondrial stress can trigger a favorable cellular response, resulting in an improved mitochondrial and nonmitochondrial adaptation, and thus maintain redox homeostasis [16] (Figure 1). As depicted in Figure 1, high-intensity exercise might induce mitochondrial stress, leading the mitochondria to emit ROS in order to facilitate adaptations and thus protect against subsequent cellular stress [17]. In case of excessive ROS production, this might lead to oxidative damage. 

From another perspective, aerobic exercise has been suggested to be beneficial in ROS-associated diseases, including cardiovascular pathologies [18]. Previous studies have demonstrated that regular and moderate exercise can enhance endothelia function by increasing the bioavailability of NO and improving redox states in subjects with pre-existing cardiovascular risk factors or diseases [19,20]. Nevertheless, a few studies also revealed conflicting results by showing that intense aerobic exercise could injure endothelia cells [21,22]. These results might be explained by the different exercise intensities and the resulting varying levels of oxidative damage.

As a potential countermeasure against excessive oxidative stress during exercise, antioxidative supplementations, which aim to protect against muscle damage and thus improve exercise performance, have been frequently discussed [4,22]. Nonetheless, many studies have indicated that large-dose antioxidant supplementation can interfere with intrinsic adaptive responses and may abolish the benefit of exercise [23,24]. These highly purified antioxidants can negatively affect ROS-mediated physiological processes through prooxidant mechanisms [22]. In a previously published meta-analysis, Stepanyan et al. [25] demonstrated that supplementation with vitamin E did not protect against exercise-induced lipid peroxidation or muscle damage. Instead of single antioxidative sources, it might be speculated that the intake of natural foods rich in antioxidants of phytochemicals (e.g., fruits and vegetables) might represent a more beneficial approach for enhancing the antioxidant status during exercise [26]. Along with their high antioxidant content, specific diets, including products such as oatmeal, dark chocolate, and mixed fruit beverages may also contain additional bioactive compounds which are not found in single-dose pharmacological antioxidant supplements but can act synergistically to reveal more beneficial effects than a single dose of antioxidant supplements [27,28]. Additionally, these compounds are more accessible than specific isolated antioxidants. Until now, few studies have investigated the clinical effects on exercise-induced oxidative stress by using a whole dietary strategy and consistent evidence from human study remains scarce.

## 2. Methods

The article search was conducted at the following electronic databases: Pubmed, Scopus and Web of Science. Searches were performed up to February 2021. The search term was developed with three segments: the first segment encompassed synonyms of diet; the second and third segments included synonyms of ROS and exercise, respectively. All segments were connected with the Boolean operator “AND”. The respective MeSH terms were used for each keyword. In order to avoid the potential bias caused by different baseline values of redox status, only the untrained, nonathlete, healthy population were included in this review. Animal models were not included.

## 3. Dietary Strategies

The majority of currently available studies addressed the effects of phenol-rich foods on exercise-induced oxidative stress, including dark chocolate [29,30,31], high-flavanol cocoa drink [32], green tea [33], mate tea [34], New Zealand blueberry smoothie [35], blueberries [36,37], grape juice [38,39], Montmorency cherry juice [40], tart cherry juice [41], oatmeal [42], avenanthramides (AVA)-rich cookie [43,44], juçara juice [45], Sanguinello cultivar red orange juice [46], and purple sweet potato leaves [47]. Frequently, the effects of dietary strategies on exercise-induced stress are evaluated within short-term [29,32,35,36,38,42,45], as well as long-term interventions [30,31,33,34,37,39,40,41,43,44,46,47]. Across all studies, there is a compelling amount of evidence suggesting that different dietary regimens are viable tools for decreasing exercise-induced oxidative stress. However, the different biomarkers of oxidative stress do not allow a direct comparison between studies. Therefore, the individual effects of these dietary strategies on different redox systems will be discussed in the following section.

## 4. Effects on Biomarkers of Exercise-Induced Oxidative Stress

High intensity exercise has repeatedly been demonstrated to induce excessive amounts of ROS, which may react with macromolecules such as proteins, lipids, and DNA [2]. To date, the in vivo detection of free radicals remains a challenge due to their short lifetime and the low rates of formation. Numerous techniques and assays have been used to measure oxidative stress production directly or indirectly. Accordingly, the included studies will be categorized according to whether the main effects observed were in ROS generation, oxidative stress markers, inflammatory markers or antioxidant activity (Table 1).

### 4.1. Effects of Dietary Interventions on Direct ROS Generation

To date, electron paramagnetic resonance (EPR) technology is the only method that can directly detect ROS generation in in vivo conditions [57]. Short-lived ROS can be added to the spin trap to form a spin-adduct that has a comparatively longer half-life to be detected using EPR spectroscopy [58]. Zeng et al. [42] revealed that consumption of AVA-rich oatmeal before high-intensity interval training (HIIT) significantly mitigates exercise-induced ROS generation compared to the control group, by using the EPR method. AVA, as one of the major components of polyphenolic amides (nonflavonoids), is considered the most important antioxidant found in oats [59,60]. Therefore, it can be speculated that the hydroxyl groups of AVA contribute to antioxidant defense through their ability to trap ROS in vitro [61]. Another assumption is that AVA can activate the nuclear factor erythroid 2-related factor 2 (NRF2) defense system against oxidative stress by attacking the α, β-unsaturated carbonyl moiety [62]. However, the underlying mechanisms for these effects are still unclear.

Indeed, only the study of Zeng et al., [42] applied direct ROS measurements using EPR technology, whereas the other experiments in this review used oxidative stress markers, inflammatory markers and antioxidant activity levels to interpret the changes in ROS production, as will be discussed below.

### 4.2. Effects of Dietary Interventions on ROS-Induced Macromolecule Damage

In the majority of studies, F2-isoprostanes, 8-isoprostanes, lipid hydroperoxides (LH), thiobarbituric acid-reactive substances (TBARS) and malondialdehydes (MDA) were used as the oxidative markers, which result from lipoperoxidation by oxidative damage. Similarly, protein carbonylation (PC) was used as a marker of protein damage, and 8-Hydroxydeoxyguanosine (8-oxodG) as a specific marker of 2′-deoxyguanosine damage after ROS attack to DNA. In this narrative review, *n* = 14 articles ([29,30,31,32,37,40,46,47,48,49,50,51,52,53]) showed the antioxidant effects diets on oxidative stress markers.

Davison et al. [29], Wiswedel et al., [32] and Allgrove et al., [30] observed the beneficial antioxidant effects of dark chocolate by detecting the plasma levels of F2-isoprostane, while Davison et al., [29] and Wiswedel et al., [32] confirmed the acute antioxidant effects of dark chocolate due to its polyphenolic properties, Allgrove et al., [30] and Taub et al., [31] showed that these beneficial effects can also be seen following long-term dietary interventions. The derivatives of catechin and epicatechin, which can both be defined as monomeric flavanols, are the major antioxidant components in cacao beans (chocolate) [63]. The acute antioxidant effects of flavanols in cocoa were evaluated by Davison et al., [29] who investigated the association between the increased plasma epicatechin levels and F2-isoprostanes and found decreased oxidative stress markers in a group given dark chocolate compared to a control group. In this study, after consuming 100 g dark chocolate or an isomacronutrient control bar, each healthy male subject cycled for 2.5 h at ~60% maximal oxygen uptake. Blood samples were analyzed at pre-exercise and immediately postexercise. Plasma F2-isoprostane, also showed a decline after ingestion of a high-flavanol cocoa drink combined with strenuous cycling exercise in the study of Wiswedel et al., [32]. 

Despite the acute antioxidant effects, Allgrove et al., [30] found that consuming 40 g of dark chocolate twice daily for two weeks significantly decreased plasma F2-isoprostane levels at exhaustion and after one hour of recovery in a prolonged exercise trial.

Differently, the study of Taub et al., [31] explored the mechanisms underlying the long-term antioxidant effects of dark chocolate by examining human muscle samples (quadriceps femoris). After consuming 20 g of dark chocolate or placebo for three months, the VO_2 max_ and total work of each sedentary subject was assessed on a stationary bicycle. After exercise, the skeletal muscle evidenced significant decreases in PC and increased glutathione (GSH) levels only in the dark chocolate group [31]. Furthermore, the protein levels of liver kinase B1(LKB1), adenosine monophosphate (AMP)-activated protein kinase (AMPK), peroxisome proliferator-activated receptor gamma coactivator 1-alpha (PGC1α), and their active forms (phosphorylated AMPK and LKB1), along with citrated synthase (CS) activity, were found significantly elevated [31]. Accordingly, the dark chocolate might activate upstream control systems and improve mitochondria performance in skeletal muscle, contributing to the improvements in maximum work achieved and VO_2 max_.

It has to be mentioned that the studies investigating the effects of cocoa had heterogeneous designs, which prevents us from making definite conclusions. Davison et al., [29] used 100 g dark chocolate (39.1 mg catechin, 96.8 mg epicatechin, 58.4 mg Dimer B2, 7.3 mg Dimer B5, 34.7 mg Trimer C and 10.5 mg tetramer D); Wiswedel et al., [32] chose a high-flavanol cocoa drink (187 mg flavan-3-ols/100 mL); Allgrove et al. [30] applied the 40 g 70% chocolate; Taub et al., [31] provided the dark chocolate at total of 20 g and ~100 calories per day. 

In addition to cocoa, other phenol-rich fruits also exhibited antioxidant effects during exercise by detecting oxidative stress markers, including blueberry [37], cherry [40] and red orange [46]. In the study by McAnulty et al., [37], participants consumed 150g of blueberries in a milkshake every day for one week prior to one session of high-intensity training in hyperthermic conditions. The results showed that the blueberry diet attenuated an increase in LH concentration caused by exercise stress but not F2-isoprostane levels, compared with a blueberry-flavored shake as a placebo [37]. Montmorency cherry juice (30 mL twice per day) was provided for one week before and 48 h after a bout of strength exercise in the study of Bowtell et al., [40]. The recovery of isometric muscle strength after high-intensity exercise was improved. PC were lower in the Montmorency cherry juice group compared with the isoenergetic fruit concentrate (placebo) group. Red oranges are a cultivar of the Citrus sinensis family which are generally rich in vitamin C, anthocyanins, and flavanones. The Sanguinello cultivar red orange juice (ROJ) was provided as the intervention diet in a study by Pittaluga et al., [46] due to the remarkable antioxidant ability of anthocyanin family, such as cyanidine-3-*O*-β-glucoside (C3G). In this elderly human trial, the intervention group (250 mL ROJ thrice a day for 4 weeks) had lower exhausted exercise-induced MDA, lower hypoxanthine/xanthine system activation, and less ascorbic acid consumed.

Purple sweet potato leaves (PSPL), as another phenol-rich diet, showed decreases in oxidative stress markers in an exercise trial [47]. Chang et al., [47] investigated the effects of a 7-day PSPL-diet on running exercise-induced oxidative stress in a nontrained, young male population. PSPL consumption significantly increased total polyphenols concentrations, and significantly decreased plasma PC and TBARS in the PSPL group [47]. PSPLs, botanically identified as Ipomoea batatas (L.) Lam, have the highest levels of polyphenols and flavonoids (33.4 ± 0.5 mg gallic acid/g and 426.8 ± 8.9 μg/g dry wt) [64]. This research group also proved that the PSPL diet could modulate antioxidative status [65] and immune responses [66] in basketball players during a training period.

Besides phenol-rich foods, probiotic-rich dairy also showed promising antioxidant effects on the levels of oxidative stress markers. Mazani et al., [48] described the antioxidant effect of 450 g of probiotic yogurt taken daily for two weeks by young females. Compared with regular yogurt, after intense physical activity, probiotic yogurt consumption significantly decreased serum levels of MDA, and some inflammatory factors (tumor necrosis factor-α (TNF-α), matrix metalloproteinase 2 (MMP2), matrix metallopeptidase 9 (MMP9)), and increased the levels of superoxide dismutase (SOD), glutathione peroxidase (GP_X_), and total antioxidant capacity (TAC). This result might be explained by the previous assumptions that some strains of probiotics positively prevent and correct oxidative stress in humans due to their direct antioxidative activity and positive effect on the immune system [67,68]. 

Lycopene is a carotenoid that is mainly found in tomatoes [69]. Among dietary carotenoids, lycopene is one of the most active antioxidants with a singlet-oxygen-quenching capacity twice as high as that of β-carotene and ten times greater than that of α-tocopherol [70]. However, the underlying mechanism of how it resists oxidative stress in vivo is still unclear. Two major potential hypotheses to explain the antioxidant abilities of lycopene are oxidative and nonoxidative mechanisms [69]. In a human trial by Harms-Ringdahl et al., [49], a daily intake of tomato juice, equal to 15 mg lycopene per day for five weeks significantly reduced the serum levels of 8-oxodG after extensive physical exercise.

A few dietary strategies that have been described as mixed foods have shown antioxidant effects on markers of oxidative stress. To investigate the effects of mixed antioxidant foods on resistance training-induced oxidative stress, a diet containing salmon flakes, green and yellow vegetable juice, and lingonberry jam, which contain astaxanthin, β-carotene, and resveratrol was provided by Kawamura et al., [50]. This mixed diet was consumed by the intervention group twice a week for 10 weeks. The results showed that serum PC levels tended to be lower immediately after exercise than before exercise in the intervention group only. The mixture of these nutrients might collectively enhance the antioxidant effects in this trial.

However, the combined antioxidant mechanisms of mixed foods are complex, and previous studies have shown inconsistent results for some mixed diets. Sureda et al., [51] demonstrated that a mixed beverage with vitamin C and E reduced the plasma oxidative damage induced by a half-marathon. After this, Carrera-Quintanar et al., [71] proved that ingestion of a mixed beverage that included an excess of polyphenolic antioxidants (*Lippia citriodora*) for 21 days could interfere with antioxidant activities and reduce the gene expression of specific enzymes (e.g., Cu-Zn-SOD, Mn-SOD and glutathione reductase (GRD)) in neutrophils, in a human study without exercise intervention. Recently, Carrera-Quintanar et al., [52] compared the antioxidant effects of two mixed beverages and one control beverage on exercise-induced oxidative damage: a mixed beverage enriched with vitamins C and E; the same beverage with extra *Lippia citriodora* extract; and the control beverage. This study was performed in a 2000-m running exercise trial. However, the results showed that all the oxidative stress markers increased in the control group, plasma PC significantly increased only in the mixed beverage with *Lippia citriodora*, and no significant changes in oxidative stress levels were detected for the mixed beverage which only added vitamins C and E. Accordingly, further studies are needed to explore the mechanisms by which certain antioxidants in the *Lippia citriodora* extract were less effective at combating oxidative stress than their components in isolation. 

In contrast, one study did not find positive effects by testing oxidative stress markers [53]. In a four-month study of 216 women, a twice-daily multi-nutrient-fortified milk drink (MFMD), containing added protein, milk fat globule membrane (phospholipids and other bioactives), vitamin D, calcium, and other micronutrients, did not enhance the effects of an exercise program on markers of oxidative stress (marker: 8-isoprostane, PC) and the primary outcome measure of stair climbing ascent power [53]. However, the MFMD did elicit greater improvements in various secondary outcomes of physical functions compared to an energy-matched placebo [53].

### 4.3. Effects of Dietary Interventions on Inflammatory Markers

Exercise-induced oxidative stress can activate a range of transcription factors that contribute to the differential expression of certain genes involved in inflammatory pathways [72]. In this review, diets with antioxidant effects have demonstrated to reduce inflammatory markers including neutrophil respiratory burst (NRB), interleukin-6 (IL-6), nuclear factor-kappa B (NF-κB), granulocyte-colony stimulating factor (G-CSF), interleukin-1 receptor antagonist (IL-1Ra), soluble vascular cell adhesion molecule-1 (sVCAM-1). In this narrative review, *n* = 3 studies ([43,44,54]) showed decreases in inflammatory markers from the diet interventions.

Koenig et al., [43] and Zhang et al., [44] investigated the eight-week effects of AVA-rich cookies on exercise-induced oxidative stress by detecting the inflammatory markers. Both found that this AVA-rich diet decreased ROS production from the NRB after high intensity downhill training when compared to control group. Additionally, plasma IL-6 and NF-κB activity significantly decreased in AVA group versus control group in the study of young women by Koenig et al., [43]. Zhang et al., [44] further found that the neutrophil stimulating cytokine G-CSF, IL-1Ra, sVCAM-1 was significantly lower in AVA group compared to the control group after exercise stress. Similar to the study by Zeng et al., [42], the main antioxidant effects of these two investigations were described as being attributed to AVA components. However, Zeng et al., [42] examined the direct ROS generation by EPR method, whereas these two studies detected indirect inflammatory markers. 

The role of vitamin C in mitigating the overproduction of ROS caused by high-intensity exercise is assumed to occur by helping to preserve the redox integrity of the immune cells and reduce the inflammation [73,74]. A four-week cashew apple juice (CAJ) supplementation was shown to enhance leukocyte count by reducing oxidative stress after high-intensity exercise in trained and untrained men [54]. The CAJ contained significant amounts of vitamin C (3.36 mg/100 g) and further antioxidants such as anacardia acids. The anacardia acids in CAJ may enhance the ability of vitamin C to prevent the generation of superoxide radicals by inhibiting xanthine oxidase and increasing heme oxygenase-1 [75]. However, the previously elaborated study by McAnulty et al., [37] compared the antioxidant effects of a blueberry diet and vitamin C supplements in hot training conditions. In contrast, the results supported the assumption of a prophylactic effect of polyphenol on exercise-induced oxidative stress, but not of vitamin C.

### 4.4. Effects of Dietary Interventions on Antioxidant Activity

In concert with alterations affecting levels of oxidative stress markers and inflammatory markers, exercise-induced oxidative stress could attenuate the endogenous antioxidant defense including enzymatic antioxidant activity (catalase (CAT), SOD, GPx, cyclooxygenase-2 (COX-2)) and nonenzymatic antioxidant activity (GSH, oxygen radical absorbance capacity (ORAC), total antioxidant capacity (TAC), total antioxidant status (TAS), ferric reducing antioxidant power (FRAP), vitamins C and E, and reduced glutathione content). In this current review, *n* = 10 articles ([33,34,35,36,38,39,41,45,55,56]) found that dietary strategies increased antioxidant activity.

Some included studies have demonstrated that phenol-rich foods could increase antioxidant capacity during high intensity exercise. As for cocoa, the major antioxidant properties in tea leaves are flavanol compounds, such as catechin and epicatechin [76]. Two studies by Panza et al., [33,34] investigated the antioxidant activity following the consumption of green tea or mate tea for one week in young men undergoing resistance exercise. Green tea increased the values of total polyphenols, GSH, FRAP and diminished the plasma levels of LH after a bench press exercise [33]. Similarly, mate tea increased the concentration of total polyphonic compounds at all time points and the levels of GSH after twenty maximal eccentric elbow flexion exercises [34].

McLeay et al. [35] and Park et al., [36] demonstrated the short-term effects of blueberries by detecting antioxidant activity. McLeay et al., [35] researched the antioxidant effects of New Zealand blueberries on exercise-induced muscle damage (EIMD) after strenuous eccentric exercise. This study showed that ingestion of a blueberry smoothie before and after EIMD accelerates recovery of muscle peak isometric strength, which might be due to the decreased ROS-generating potential and the gradual increase in plasma antioxidant capacity [35]. Similar results were reported by Park et al., [36]—increased TAS levels and significantly decreased IL-6 and C-reactive protein (CRP) levels were found in the blueberry supplementation period following exercise. Meanwhile, VO_2_ max and exercise performance time were grown during the blueberry supplementation period.

Integral grape juice was used as the dietary strategy against exercise-induced oxidative stress in an acute study [38] and a 28-day study [39] by Toscano et al. A single-dose grape juice (10 mL/kg/day) taken 2 h before running to exhaustion showed an ergogenic effect by significantly increasing TAC at the postexercise time point compared to the baseline level [38]. After taking the same daily dose for 28 days, the grape juice group exhibited significant increases in plasma levels of TAC, vitamin A and uric acid compared to control group [39]. These improvements in antioxidant capacity found in both studies were accompanied by an increased time to exhaustion in recreational runners [38,39].

Tart cherry juice showed subchronic positive effects on antioxidant activity caused by high-intensity exercises in the study of Howatson et al. [41]. Tart cherry juice (two 8 oz bottles per day) was given for five days before, on the day of, and for 48 h following, a marathon run [41]. In the study of tart cherry juice, significantly increased TAS levels, and significantly reduced inflammation (IL-6, CRP, uric acid) and MDA levels were found in the intervention group compared with the placebo group [41]. One 8 oz bottle of tart cherry juice, which contains the equivalent of 50–60 cherries, provided at least 600 mg of phenolic compounds [41]. 

Juçara juice (*Euterpe edulis Martius*), with a similar chemical composition to açai fruit (*Euterpe oleracea Martius*), has strong antioxidant activity due to its high anthocyanins content [77]. Copetti et al. [45] evaluated the acute antioxidant effect of juçara juice during HIIT by observing antioxidant status. The HIIT was performed 1 h after drinking 250 mL of juçara juice or water (control). Compared to the control group, juçara juice intake promoted a decrease in oxidative stress index (OSI) immediately post exercise and an increase in reduced glutathione 1 h after exercise [45]. OSI was defined as the ratio of serum total oxidant status (TOS) to serum TAC in this study. These enhancements came with a significant increase in total plasma phenols content [45]. 

In addition to phenols-rich foods, Iwasa et al., [55] found that fermented milk (Lactobacillus helveticus) inhibited the reduction of antioxidant capacity (ORAC assay) induced by acute resistance exercise in a clinical trial. In the processing and manufacturing of fermented milk, *Lactobacillus* digests the proteins and transforms them into small peptides, which are more readily absorbed by the intestines than amino acids or large oligopeptides. Although the evidence for potential mechanisms is still lacking, the small peptides might contribute to the increasing level of antioxidants in contracting muscles [55]. 

Nevertheless, one of the dietary strategies included in this review showed no antioxidant effects. Beavers et al., [56] found that soy foods, as a source of high-quality protein and isoflavones, did not elevate antioxidant capacity (GPx, COX-2) after high intensity exercise stress. 

In summary, the included studies elucidated the antioxidant effects of different dietary strategies by detecting ROS generation, oxidative stress markers, inflammatory markers and antioxidant activity. Among them, most studies included in this narrative review found that phenol-rich foods reduced exercise-induced oxidative stress, by short-term consumption [29,32,35,36,38,42,45] or long-term intake [30,31,33,34,37,39,40,41,43,44,46,47]. The potential antioxidant ability of dietary polyphenols has been widely demonstrated in both in vitro and in vivo studies [78]. As secondary plant metabolites, the majority of polyphenols have at least one aromatic ring and typically occur in the form of glycosides in their molecules. According to the chemical structures of the aglycones, polyphenols have been classified into flavonoid polyphenols (e.g., flavanols, anthocyanidins) and nonflavonoid polyphenols (e.g., phenolic acid, polyphenolic amides (e.g., AVA), resveratrol, curcumin, ellagic acid) [78]. Over 8000 polyphenolic compounds have been identified and more than 4000 flavonoids have been found among them. The functional hydroxyl group (OH) of polyphenolic compounds is assumed to play a key role in antioxidant defense [78]. It may inhibit the ROS synthesis, chelate with trace elements responsible for ROS generation, scavenge excessive ROS production, and improve the antioxidant defense [2,79]. The Phenol-Explorer Database (www.phenol-explorer.eu, accessed on 11 February 2021)) offers data on the presence of 502 polyphenols in 452 foods and provides an analysis of the volume of polyphenols included in a food serving [80]. From the database, the 100 richest dietary sources of polyphenols were identified [80]. The main rich sources of polyphenols are cocoa, fruits, vegetables, whole grains and tea in this review [78,80,81,82]. Besides flavanols, another category of flavonoid polyphenols, plant anthocyanidins, are found in the red, blue, and purple pigments of a plurality of flower petals, vegetables, fruits and some special types of grains (e.g., black rice). In this review, blueberries, grapes, cherries, and citrus fruits are all the main fruit sources. In addition to AVA, other nonflavonoid compounds have shown potential antioxidant effects in vitro [78]. Capsaicin, mainly found in chili peppers, is another polyphenolic amide compound that belongs to the nonflavonoids group [83]. Curcumin is a potent antioxidant in turmeric [84]. Resveratrol is a unique component of red wine and grapes [78]. Lignans are present in bound forms in sesame, flax and several grains [78]. Ellagic acid and its derivatives are contained in berry fruits (e.g., strawberries and raspberries) and the skins of some different tree nuts [78]. However, the mechanisms of their antioxidant effects in vitro and in vivo are still unclear. 

A potential limitation of this narrative review needs to be mentioned. It is reported that endurance training could influence the oxidative stress response to acute exercise [85]. Variations in training type, intensity, and duration can activate different patterns of oxidant–antioxidant balance, resulting in different transcriptome responses for regulatory and metabolic processes [85,86]. In order to avoid the potential bias caused by different baseline values of redox status, only the untrained general population were included in this review. 

When interpreting the results of diet interventions on exercise-induced oxidative stress, it is important to note that some studies included methods with questionable validity. One such method includes the assessment of TBARS, which was commonly regarded as a quantification assay for lipid peroxidation [87]. This assay, however, is no longer recommended in redox research, since TBA-reactive material in human body fluids is not related to lipid peroxidation [87]. TBARS lacks specificity since it reacts with numerous substrates in the assay medium to form MDA. Thereby, most MDA is produced artificially [88]. Another assay which is equally flawed is the measurement of total antioxidant capacity (TAC) [89]. TAC, for instance, is greatly dependent on plasma albumin or urate levels [87] and therefore, exercise-induced changes in urate concentrations can bias the assay by urate reacting with the peroxyl radical [88]. 

The only method that can directly measure free radicals is electron paramagnetic resonance (EPR), because it identifies the presence of unpaired electrons. However, EPR alone is limited to detecting only fairly unreactive radicals, since highly reactive compounds do not accumulate to measurable levels [87]. Therefore, specific agents (e.g., spin traps or spin probes) have been developed which allow forming a more stable radical which can then be detected by EPR [57,87]. Future studies are thus needed which combine valid direct and indirect measures of oxidative stress in order to further investigate the effects of different dietary strategies on exercise-induced oxidative stress.

## 5. Perspectives

The biological actions of antioxidant properties from an antioxidant-rich diet are complex. Previous studies have led to a few contrasting results, probably due to differences in antioxidant composition and actual bioavailability. Moreover, only Zeng et al., [42] used a direct measurement technique for assessing ROS generation, whereas the majority of studies used indirect markers of oxidative stress as a surrogate marker. Furthermore, the available studies show a high level of heterogeneity in their study designs. Consistent and standardized research procedures may be essential to obtain convincing evidence in future studies.

In this narrative review, most studies found positive effects of dietary strategies on exercise-induced ROS generation. Especially, phenol-rich diets showed effects in combating exercise-induced oxidative stress in the greater proportion of the articles. Accordingly, while dietary strategies might help to keep ROS generation in a physiological range during exercise, the use of the antioxidant-rich diets may upregulate the endogenous antioxidants’ defense system, which may have important implications for preventing excessive damage and facilitating recovery. Nevertheless, consistent evidence is still lacking, and the underlying mechanisms in human trials are not well understood. 

In future research, antioxidant dietary regimens for different individuals should developed with consideration of individual physiological characteristics and style. Moreover, a standardized assay as well as a study design protocol needs to be established. Further research is necessary to explore optimal antioxidant diets and to elucidate the potential mechanisms, by using standard detection assays and research protocols.

## 6. Conclusions 

Although the literature about the effects of whole dietary strategies on exercise-induced oxidative stress is still scarce, the majority of the studies demonstrated favorable effects. Within this context, most of the included studies showed that phenol-rich foods had positive effects on exercise-induced oxidative stress in short-term and long-term experimental designs. Nevertheless, the protocols are still very heterogeneous and further systematically designed studies are needed to strengthen the evidence.

## Figures and Tables

**Table 1:** Effects of dietary strategies on exercise-induced oxidative stress.

Main Result Category	Study	Type of Diet	Nutritional Protocol	Type and Intensity of Exercise	Detection Method			
					ROS Generation	Oxidative Stress Marker	Inflammation Marker	Antioxidant Activity
ROS Generation	Zeng et al. [42]	Oatmeal	Oat flake + skim milk versus Fasting; 2 h before exercise	Body weight HIIT, 30 min	↓	N/A	N/A	N/A
ROS-induced Macromolecule Damage	Davison et al. [29]	Dark chocolate	Dark chocolate versus cocoa-liquor-free control bar versus neither, 2 h before exercise	Cycling, 2.5 h	N/A	F2-isoprostane↓	Circulating leucocyte↔, IL-6↔	N/A
	Wiswedel et al. [32]	High-flavanol cocoa drink (HFCD)	HFCD versus low-flavanol cocoa drink (LFCD), 2 h before exercise	Cycling, 29 min	N/A	F2-isoprostane↓	N/A	N/A
	Allgrove et al. [30]	Dark chocolate	Dark chocolate versus isocarbohydrate-fat control cocoa-liquor-free chocolate, twice/d, 2 weeks	Cycling for 90 min followed by 25 min exhaustion time trial	N/A	F2-isoprostane↓	Circulating leucocyte↔, IL-6↔, IL-10↔, IL-1Ra↔	N/A
	Taub et al. [31]	High-flavanol dark chocolate (HFCHO)	HFCHO versus Low-flavanol dark chocolate (LFCHO), 3 months	Ramped exercise on stationary bicycle (Cardiopulmonary exercise testing), ~10 min	N/A	PC↓	N/A	GSH/GSSH↑
	McAnulty et al. [37]	Blueberry	Blueberries versus blueberry-flavored shake, 7 days	Running, until a core temperature of 39.5 °C was reached	N/A	LH↓, F2-isoprostanes↔	IL-6↔, IL-8↔, IL-10↔	FRAP↔
	Bowtell et al. [40]	Montmorency cherry juice	Montmorency cherry juice versus isoenergetic fruit concentrate, 7 d before and 48 h after exercise	Two trials of 10 sets of 10 single-leg knee extensions	N/A	PC↓	N/A	N/A
	Pittaluga et al. [46]	Fresh red orange juice (ROJ)	ROJ versus nothing extra, thrice/day, 4 weeks	A single bout of exhaustive exercise by cycle ergometer (3 min warm-up, an initial load of 25 W, and further increments of 15 W/3 min)	N/A	MDA↓, ascorbic acid↓, hypoxanthine/xanthine↓	N/A	N/A
	Chang et al. [47]	Purple sweet potato leaves (PSPL)	Standard cooked PSPL versus low-polyphenols diet, 7 days	Treadmill running at 70% VO_2max_, 1 h	N/A	PC↓	IL-6↓, HSP72↔	TAC (FRAP assay)↑, polyphenols↑
	Mazani et al. [48]	Probiotic yoghurt	Probiotic yoghurt versus ordinary yoghurt, 2 weeks	Exhaustive exercise (Bruce test)	N/A	MDA↓	TNF-α↓, MMP2↓, MMP9↓	SOD↑, GPX↑, TAC↑,
	Harms-Ringdahl et al. [49]	Tomato juice	Tomato juice versus nothing extra, 5 weeks	Cycle ergometer at 80% of HRmax, 20 min	N/A	8-oxodG↓	N/A	N/A
	Kawamura et al. [50]	Mixed diet	Salmon flakes + green and yellow vegetable juice + lingonberry jam versus normal diet, 10 weeks	Resistance training twice/week, 10 weeks	N/A	PC↓	N/A	N/A
	Sureda et al. [51]	Mixed beverage	Almond-based isotonic and energetic beverage with vitamin C and E versus Nonenriched beverage, 1 month	A half-marathon race (21 km-run)	N/A	MDA↓	N/A	N/A
	Carrera-Quintanar et al. [52]	Mixed beverage	Mixed beverage with extra *Lippia citriodora* extract versus mixed beverage enriched with vitamins C and E, 22 days	2000-m running exercise trial	N/A	PC↑	N/A	SOD↓, GRD↓
	M Daly et al. [53]	Multinutrient-fortified milk (MFMD)	MFMD versus placebo milk, twice/d, 4 months	Resistance exercise 3 d/week, 4 months	N/A	PC↔, 8-isoprostane↔	N/A	N/A
Inflammatory Markers	Koenig et al. [43], Zhang et al. [44]	AVA-enrich cookies	4.6 mg AVA/cookie versus 0.2 mg AVA/cookie, 2 cookies/day, 8 weeks	Downhill running, 1 h	N/A	N/A	NRB [43]; NF-κB↓ and IL-6 [43]; G-CSF, IL-1Ra and sVCAM-1 [44]	N/A
	Prasertsri et al. [54]	Cashew apple juice (CAJ)	CAJ versus placebo (isocaloric), 4 weeks	Cycling at 85% of VO_2max_, 20 min	N/A	MDA↓, 8-isoprostane↓,	Leukocyte count↑	N/A
Antioxidant Activity	Panza et al. [34]	Mate tea	Mate tea versus water, 11days, exercise and blood test were performed at 8th day	Three sets of twenty maximal eccentric elbow flexion exercises	N/A	N/A	N/A	GSH↑, GSSG↔, GSH/GSSG↔, LOOH↔
	Panze et al. [33]	Green tea	Green tea versus Water, three times/day, 7 days	A bench press exercise, four sets, 10 to 4 repetitions	N/A	LH↓	N/A	TAC (FRAP assay)↑, total polyphenol↑, GSH↑
	McLeay et al. [35]	New Zealand blueberry	Blueberry + banana + commercial apple juice versus Shake dextrose + banana + commercial apple juice (isocaloric); 5 and 10 h pre, immediately, 12 and 36 h after exercise	3 sets × 100 eccentric repetitions of quadriceps muscle	N/A	PC↓	IL-6↓	TAC (FRAP assay)↑, ROS-GP↓
	Park et al. [36]	Blueberry	Blueberry + aronia + sugar + refined water versus nothing extra, before exercise	Treadmill exercise (Bruce test)	N/A	N/A	IL-6↓, CRP↓	TAS↑
	Toscano et al. [38,39]	Grape	Integral grape juice versus isocaloric, isoglycemic and isovolumetric control beverage, 10 mL/kg/day, 2 h before exercise [38], or for 28 days [39]	Time-to exhaustion exercise test, anaerobic threshold test and aerobic capacity test	N/A	N/A	N/A	TAC↑ [38,39], UA↑ and vitamin A↑ [39]
	Howatson et al. [41]	Tart cherry	Tart cherry juice versus control, before, on the day of, and 48 h following exercise	A marathon run	N/A	MDA↓	IL-6↓, CRP↓, UA↓	TAS↑
	Copetti et al. [45]	Juçara (*Euterpe edulis Martius*)	Juçara juice versus Water, 1 h before exercise	HIIT, 17 min	N/A	N/A	N/A	OSI↓
	Iwasa et al. [55]	Fermented milk	Fermented milk (*Lactobacillus helveticus*) versus equivalent dose of unfermented milk, 1 h before and 2 h after exercise	Resistance exercise consisting of five sets of leg and bench presses	N/A	N/A	hsCRP↓, TNF-a↔	TAC (ORAC assay)↑
	Beavers et al. [56]	Soy	Soy versus dairy milk, 3 serving/d, 4 weeks	Downhill-running at 60% VO_2max_ and −10% grade, 45 min	N/A	N/A	TNF-α↔, IL-1β↔, IL-6↔	GPx↔, COX-2↔

Legend: the arrows represent increase (↑), decrease (↓), no change (↔). Abbreviation list: high intensity interval training (HIIT), lipid hydroperoxide (LH), malondialdehyde (MDA), protein carbonyls (PC), superoxide dismutase (SOD), tumor necrosis factor-α (TNF-α), interleukin-6 (IL-6), interleukin-8 (IL-8), interleukin-10 (IL-10), interleukin-1 receptor antagonist (IL-1Ra), nterleukin-1β (IL-1β), matrix metalloproteinase 2 (MMP2), matrix metalloproteinase 9 (MMP9), neutrophil respiratory burst (NRB), nuclear factor-kappa B (NF-κB), total antioxidant status (TAS), Avenanthramides (AVA), soluble vascular cell adhesion molecule-1 (sVCAM-1), granulocyte-colony stimulating factor (G-CSF), lipid hydroperoxides (LOOH), 8-hydroxy-2′-deoxyguanosine (8-oxodG), ferric reducing ability of plasma (FRAP), glutathione (GSH), glutathione/oxidized glutathione (GSH/GSSH), uric acid (UA), radical oxygen species-generating potential (ROS-GP), heat shock proteins (HSP72), oxidative stress index (OSI), glutathione peroxidase (GPx), cyclooxygenase-2 (COX-2), total antioxidant capacity (TAC), glutathione reductase (GRD), C-reactive protein (CRP).
